# Identification and profiling of microRNAs during sheep’s testicular development

**DOI:** 10.3389/fvets.2025.1538990

**Published:** 2025-03-31

**Authors:** Binpeng Xi, Xuejiao An, Yaojing Yue, Haimiao Shen, Gaohui Han, Yanan Yang, Shengguo Zhao

**Affiliations:** ^1^College of Animal Science and Technology, Gansu Agricultural University, Lanzhou, China; ^2^Key Laboratory of Animal Genetics and Breeding on the Tibetan Plateau, Ministry of Agriculture and Rural Affairs, Lanzhou Institute of Husbandry and Pharmaceutical Sciences, Chinese Academy of Agricultural Sciences, Lanzhou, China; ^3^Sheep Breeding Engineering Technology Research Center of Chinese Academy of Agricultural Sciences, Lanzhou, China; ^4^Dongxiang County Mutton Sheep Industry Research Center, Linxia, China; ^5^Dongxiang County Animal Husbandry Development Center, Linxia, China

**Keywords:** testicular development, Southdown × Hu sheep hybrid F1 generation, microRNA, spermatogenesis, small RNA sequencing

## Abstract

The normal development of the testis is essential for male reproduction, as it is the site of sperm production and a prerequisite for spermatogenesis. MiRNAs play crucial roles in various testicular biological processes, including cell proliferation, spermatogenesis, hormone secretion, metabolism, and reproductive regulation. In this study, we utilized deep sequencing data to analyze the expression patterns of small RNAs in testicular tissues of Southern × Hu sheep F1 hybrids at 0, 3, 6 months, and 1 year of age, thereby exploring the functions of miRNAs in testicular development and spermatogenesis. A total of 787 known miRNAs and 415 novel miRNAs were identified. We identified 217, 254, 405, 130, 305, and 138 DE miRNAs in the testes of M0 vs. M3, M0 vs. M6, M0 vs. Y1, M3 vs. M6, M3 vs. Y1, and M6 vs. Y1, respectively. GO annotation and KEGG pathway analysis of DE miRNA target genes revealed that target genes such as *YAP1*, *ITGB1*, *DOT1L*, *SMAD4*, and *SOX9* may be involved in various biological processes, including reproductive pathways such as FOXO, Hippo, Wnt, cAMP, Rap1, and MAPK signaling pathways. The expression levels of 12 randomly selected miRNAs in testes at 0, 3, 6 months, and 1 year of age were detected by qRT-PCR, and the results were consistent with the sequencing data. This study characterized and investigated the differential expression of miRNAs in sheep testes at different developmental stages using deep sequencing technology. These findings will contribute to a deeper understanding of the functions of miRNAs in regulating testicular development and enhancing reproductive performance in male sheep.

## Introduction

Hu sheep are mainly produced in Jiaxing and Taihu Lake regions of Zhejiang Province. As a unique breed of multi-parous sheep in our country, Hu sheep has many advantages such as perennial estrus, early sexual maturity, strong adaptability and suitability for crossing (1). The Southdown sheep, a short-wooled meat breed, is characterized by its dense, short, and light fleece, early maturity, ease of fattening, and tender meat, making it a preferred choice for crossbreeding sires (2). The enhancement of animal performance can be achieved through the introduction of breeds for crossbreeding improvement, a widely adopted strategy in animal production. Cross breeding can also combine the excellent characteristics of multiple varieties, create new characteristics that the original parents do not have, and enhance the vitality of the offspring (3). The Southdown × Hu F1 hybrids enhanced meat quality and increased lambing rates, retaining the high productivity of Hu sheep while inheriting the superior meat yield, quality, and rapid growth traits of Southdown sheep (4). As an important reproductive organ of male animals, the testis is mainly responsible for the production of sperm and androgens (5). Because normal testicular development is essential for breeding, studying testicular development in sheep is crucial for enhancing semen quality and increasing lamb production.

MicroRNA (miRNA) is a non-coding RNA between 18 and 24 nucleotides in length, which controls post-transcriptional gene silencing by interacting with the 3′-untranslated region (3′-UTR) of target mRNA, stimulating mRNA degradation or blocking translation (6). In the past few decades, many studies have investigated miRNA expression profiles using microarray technology, small RNA sequencing and reported that many miRNAs are expressed in mouse and human germ cells. In mammals, miRNAs are key controllers of cell differentiation and function, mediate a variety of cellular processes, and play an indispensable role in testicular development and spermatogenesis (7). Substantial evidence indicates that microRNAs (miRNAs) play pivotal roles in various facets of reproductive physiology, encompassing testicular development, spermatogenesis, and the regulation of functional effects (8–10). In the study of miRNA in Tibetan sheep, 1,118 immune-related miRNAs were found and speculated to be related to testicular development and meiosis in Tibetan sheep. Similarly, a recent study showed that miR-301b-3p and miR-3584-5p could promote the proliferation of rat immature testicular Sertoli cells by targeted inhibition of *RASD1* gene expression (11). Yu et al. (12) found in mice that miR-34c could promote the differentiation and meiosis process of Spermatogonial stem cells (SSCs) by targeting *Nanos2* gene and up-regulating the expression of meiosis-related genes *Stra8* and *Dazl*. Smorag et al. (13) found that miR-34b-5p could regulate the meiotic process of mouse spermatocytes by targeting *IGFBP2* gene. Overexpression of miR-10a in human and mouse testicular germ cells can target and inhibit the expression of *Rad51* gene, leading to meiotic arrest and complete male infertility (14). Zhang et al. (15) found that miR-34b could target *MAP2K1* (also known as *MEK1*) to induce apoptosis of bovine testicular Sertoli cells through MEK/ERK signaling pathway. However, miRNAs involved in testicular development and their underlying molecular mechanisms have not been identified in Southdown × Hu F1 hybrids.

In this study, we used deep sequencing technology to characterize and study the differential expression of miRNAs in the testis of sheep at different stages of development, understand the molecular regulation mechanism, and identify the key miRNA targets involved in sheep testicular development and spermatogenesis. This study will help to further understand the function of miRNA in sheep testicular development and to identify key miRNAs that enhance reproductive performance in male sheep in the future.

## Materials and methods

### Animals and sample preparation

Twelve healthy Southdown × Hu F1 sheep were castrated in Qinghuan Mutton Sheep Breeding Company in Gansu Province. The age of the sheep was obtained from sheep breeding records. There were 3 sheep at 0 months old (newborn, namely M0–1, M0–2, M0–3), 3 sheep at 3 months old (sexually immature, namely M3–1, M3–2, M3–3), 3 sheep at 6 months old (sexually mature, namely M6–1, M6–2, M6–3), and 3 sheep at 1 year old (adult, namely Y1–1, Y1–2, Y1–3). We removed testes from 12 sheep after anesthesia and then stored them in an RNA/DNA sample protector (Servicebio, Wuhan, China). The testis of each sheep was dissected longitudinally, and the right testicular tissue of each sheep was collected, part of which was immediately frozen in liquid nitrogen and stored at −80°C for total RNA and protein extraction. The remaining portion will be fixed in a 2.5% glutaraldehyde solution and subsequently processed for paraffin embedding and sectioning.

### Small RNA library construction and sequencing

After total RNA was extracted by Trizol reagent kit (Invitrogen, Carlsbad, CA, United States), the RNA molecules in a size range of 18–30 nt were enriched by polyacrylamide gelelectrophoresis (PAGE). Then the 3′ adapters were added and the 36–48 nt RNAs were enriched. The 5′ adapters were then ligated to the RNAs as well. The ligation products were reverse transcribed by PCR amplification and the 140–160 bp size PCR products were enriched to generate a cDNA library and sequenced using Illumina HiSeq Xten by Gene DenovoBiotechnology Co. (Guangzhou, China)

### Bioinformatics analysis

Basic reads are converted to sequence raw data by base calling. Low-quality reads were filtered to remove reads containing 5′ primer contaminants and poly (A). Reads without 3′ adapters and insert tags, as well as Reads shorter than 15 nt or longer than 41 nt in the raw data, were filtered out to obtain clean Reads. For preliminary analysis, the length distribution of clean sequences in the reference genome was determined. Non-coding RNAs are labeled as RNAs, tRNAs, small nuclear RNAs (snRNAs), miRNA, gene, rep, miRNA and unannotation. These RNAs were aligned and subsequently Bowtie (16) searches were performed against Rfam v.10.1^1^ (17). Known miRNAs were identified by aligning sequencing reads to the miRBase v22 database^2^ and the sheep reference genome (Qar Rambouillet v1.0). The known miRNA expression patterns in different samples were analyzed (18). Subsequently, the unannotated reads were analyzed by mirdeep2 to predict novel miRNAs (19). The hairpin structure of pre-miRNA and the miRBase database were used to identify the corresponding miRNA star sequence and miRNA mature sequence. The expression of known and novel miRNAs was analyzed using TPM (transcripts per million; miRNAs normalized by TPM) (20). Differential expression analysis of miRNAs between the two groups was performed using the DEG algorithm in the R package (21). The P- values were adjusted by the method of Benjamini and Hoch-berg to control the false discovery rate (FDR). Differentially expressed miRNAs were defined when the adjusted FDR was <0.05. In addition, we used the mean TPM value to calculate fold change (FC) between groups, defining up-regulated (log2FC ≥ 1) and down-regulated (log2FC ≤ 1) miRNAs, respectively. The target of DE miRNA was predicted using Miranda software with the following parameters: single residue pair matching score ≥ 150, ΔG ≤ −30 kcal/mol, and strict 5′ seed pairing required (22). Considering the hypergeometric distribution, R was used for Gene ontology (GO) enrichment and Kyoto Encyclopedia of Genes and Genomes (KEGG) pathway enrichment analysis of DE miRNA-target-get genes (23–25).

### Validation of RNA sequence data

Twelve known sheep miRNAs were randomly selected for analysis, and qRT-PCR was used to validate the RNA-seq data. Real-time PCR was performed using a LightCycler 480 II real-time PCR apparatus (Roche, Swiss). Each sample was analyzed in triplicate. microRNA specific primer sequences were designed and synthesized in the laboratory by Qingdao Biotechnology Company based on microRNA sequences obtained from the miRBase database (Release 20.0). Primer information is given in Supplementary Table S1.

## Results

### Overview of sequencing results

**Figure 1:**
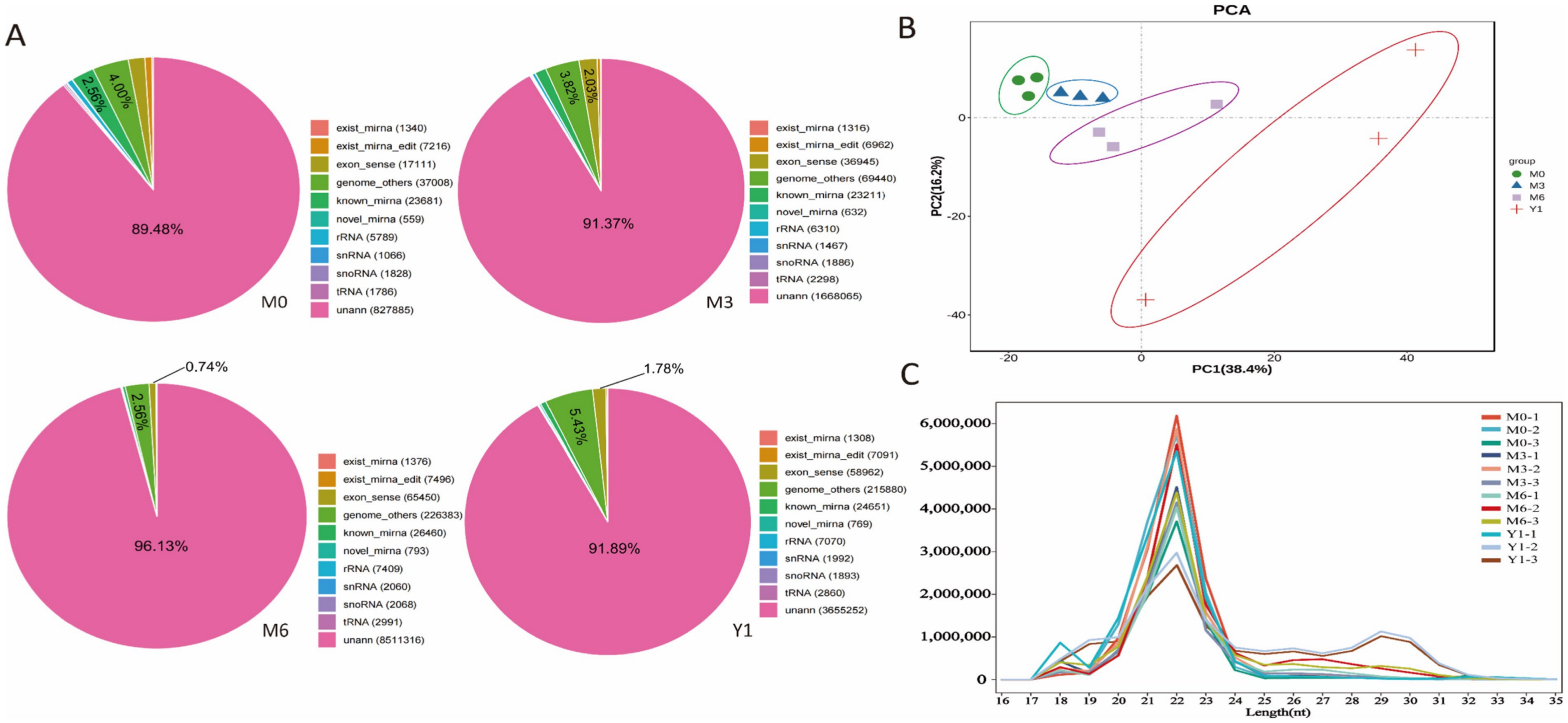
Identification and classification of miRNA in sheep testis. **(A)** Total number of unique sequences in libraries M0, M3, M6, and Y1. **(B)** PCA analysis of all mapped genes in M0, M3, M6, and Y1 groups. **(C)** Length distribution and abundance of sequences in the M0, M3, M6, and Y1 libraries.

We used Illumina Hiseq 2500 sequencing to analyze small RNA populations in 12 libraries obtained from testis of 0-month-old (M0), 3-month-old (M3), 6-month-old (M6), and 1-year-old (Y1) sheep. The results of miRNA sequence quality control are presented in Supplementary Table S2. A total of 157.27 Mb raw reads were obtained. After removing low-quality sequences, aptamers and discarding sequences shorter than 18 nt, 35.58 Mb, 32.67 Mb, 34.01 Mb and 41.01 Mb clean reads were obtained from M0, M3, M6 and Y1 libraries, respectively, for further analysis. 922,960, 1,780,035, 3,372,847, and 4,070,428 unique srRNAs were extracted from M0, M3, M6, and Y1 testes and mapped to the sheep reference genome (Supplementary Table S3). All clean reads were aligned to the miRBase database and recorded as one of the known RNA categories based on their biogenesis and annotation (Figure 1A). As shown in Figure 1A, Y1(7.21%) accounted for the largest proportion of known miRNAs, while M0(6.56%) and M3(5.85%) accounted for the second, and M6(3.3%) accounted for the smallest. However, the highest proportion of unannotated small RNAs was found in the M6(96.13%) and Y1(91.89%) libraries, which represent other types of small RNAs such as piRNAs. A principal component analysis (PCA) of all mapped genes showed that M0, M3, M6, and Y1 group could be distinguished by part along the axis of the first principal component (Figure 1B). Analysis of the read length distribution of all small RNA libraries showed that the dominant length of small RNAs was 22 nt, accounting for at least 37.72% (Figure 1C).

### Identification of known and novel miRNAs in sheep testes

**Figure 2:**
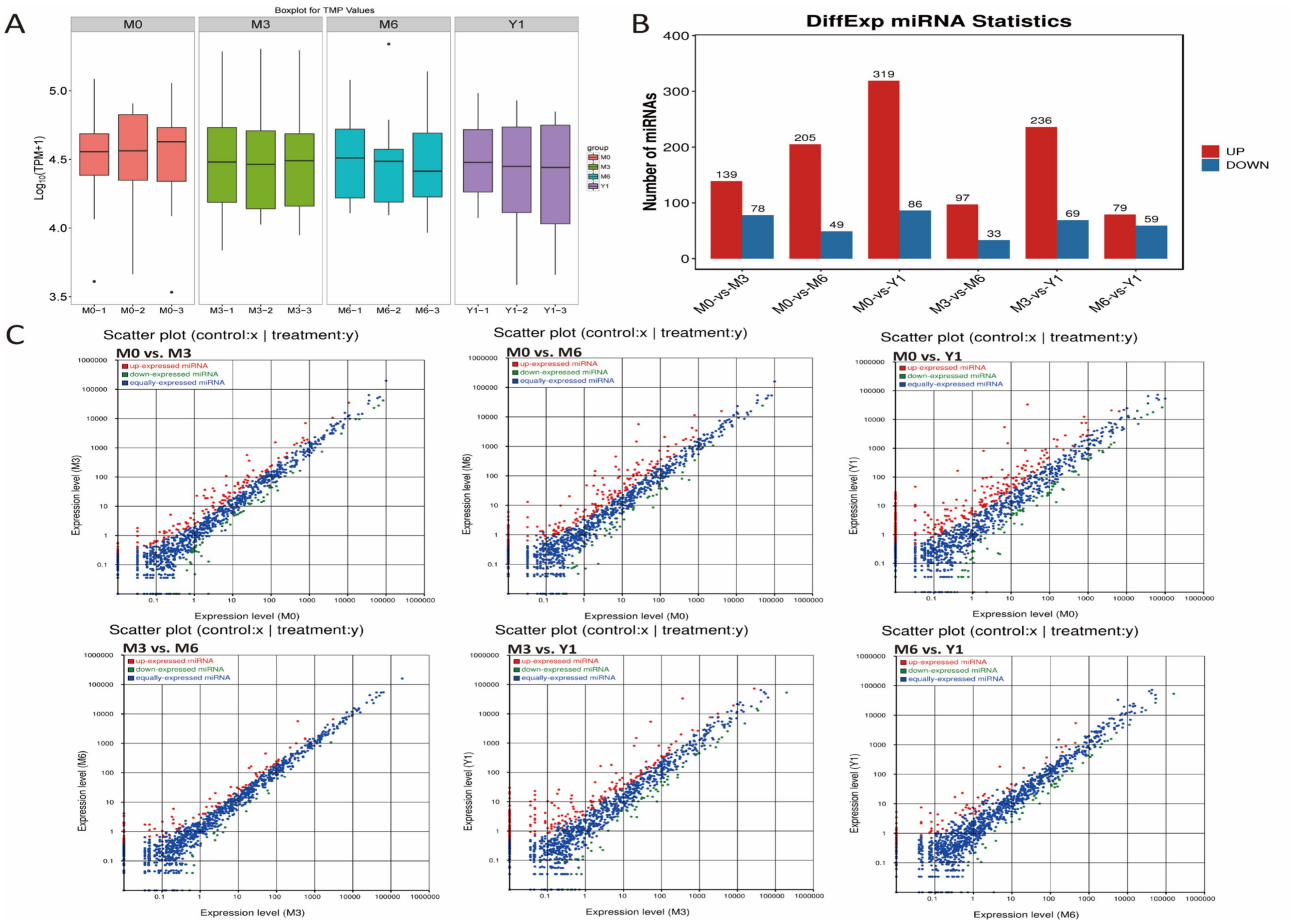
Comparative analysis of testicular miRNAs in sheep at different developmental stages. **(A)** miRNA expression levels in testis of 0, 3, 6 months and 1 year old sheep. **(B)** Barplot showing the number of up-and down-regulated DE miRNAs. **(C)** Scatter plot for differential comparison of miRNAs between pairs of groups; red, green, and blue dots indicate significantly up-regulated, down-regulated, and unchanged transcripts, respectively.

To identify miRNAs in sheep testes, the sequences obtained after removal of small RNAs, including tRNA, snRNA, and rRNA, were compared with the miRBase database to obtain known miRNAs. Sequences that were not annotated into the miRBase database were predicted as novel miRNAs. A total of 787 known miRNAs and 415 novel miRNAs were identified across the 12 libraries (Supplementary Table S4). The frequency of expression of each miRNA in the 12 libraries varied widely, ranging from a few to hundreds of thousands of sequence reads. The expression of these novel miRNAs was very low, ranging from 18 to 25 nt in length with a distribution peak of 22 nt. Overall, the expression of most known miRNAs was highest in the testis of 3-month-old sheep, followed by 6-month-old and finally 0-month-old sheep (Figure 2A). The most highly expressed members in all libraries were members of the mir-199-x, mir-125-x, miR-99-z, miR-151-x, and miR-202-x families, each with more than 100,000 reads.

### Differential expression of miRNAs in sheep testis tissue

Using the differential expression genes (DEGs) algorithm from an R package, the transcriptional changes of microRNAs (miRNAs) during sheep testis development were analyzed. Comparisons of miRNA expression profiles between different libraries were presented in Supplementary Tables S5–S10, visualized through scatter plots. Additionally, a scatter plot illustrated the pattern of DE miRNAs among all control groups, revealing that only 138 DE miRNAs were identified between M6 and Y1 testes, albeit with low expression levels (Figure 2B). Between M0 and M3, a total of 217 DE miRNAs were identified, with 139 upregulated and 78 downregulated. Between M0 and M6, 254 DE miRNAs were found, comprising 205 upregulated and 49 downregulated. From M0 to Y1, a remarkable change of 405 DE miRNAs was observed, with 319 upregulated and 86 downregulated. During the transition from M3 to M6, 130 DE miRNAs were identified, of which 97 were upregulated and 33 were downregulated. Lastly, between M3 and Y1, 305 DE miRNAs were detected, with 236 upregulated and 69 downregulated (Figure 2C).

### Prediction of target genes for DE miRNAs

Using the Miranda software, the target genes of miRNAs were predicted. Subsequently, the potential functional roles of these differentially expressed miRNAs were investigated. Potential targets for the differentially expressed miRNAs were screened based on criteria of a minimum single-residue pair total match score of ≥150 and a total energy of ≤ − 30 kcal/mol. Among the 217, 254, 405, 130, 305, and 138 miRNAs identified between M0 vs. M3, M0 vs. M6, M0 vs. Y1, M3 vs. M6, M3 vs. Y1, and M6 vs. Y1, respectively, 5,233, 8,769, 23,588, 696, 8,021, and 180 targets were predicted in 885, 1,679, 2,679, 388, 2006, and 171 target genes, respectively. For the majority of differentially expressed miRNAs, multiple distinct target genes were present; however, for some differentially expressed miRNAs, only a single target gene was identified. Additionally, certain target genes were targeted by multiple differentially expressed miRNAs. For instance, oar-miR-431 was predicted to target 914 genes, miR-199-y was predicted to target 1,510 genes, while *BMP4* was uniquely targeted by miR-142-y.

### GO enrichment and KEGG pathway analysis of target genes

**Figure 3:**
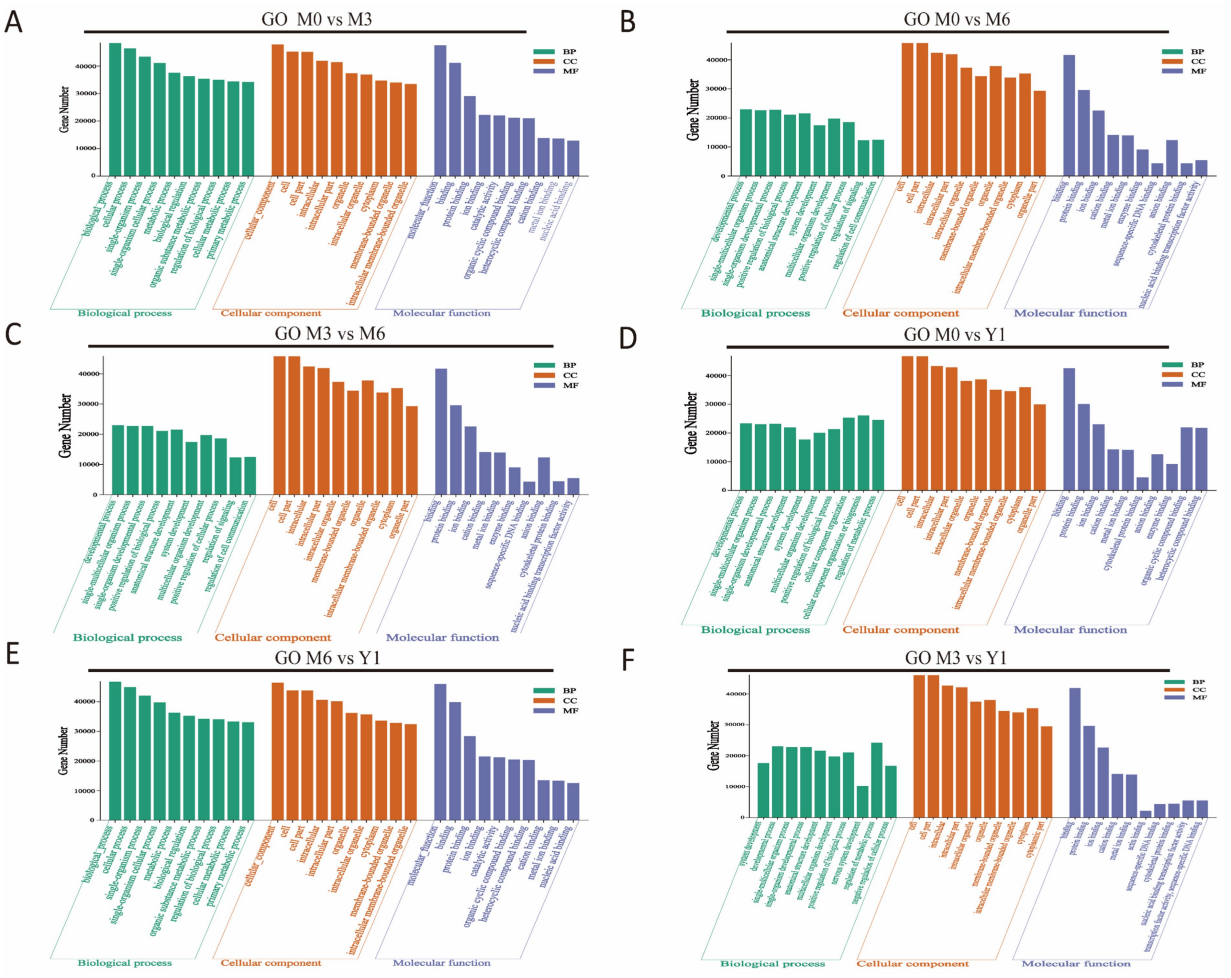
GO enrichment analysis of target genes of differentially expressed miRNAs. **(A)** M0 vs M3; **(B)** M0 vs M6; **(C)** M3 vs M6; **(D)** M0 vs Y1; **(E)** M6 vs Y1; **(F)** M3 vs Y1; M0 represents 0 months of age, M3 represents 3 months of age, M6 represents 6 months of age, and Y1 represents 1 year of age.

To gain a deeper understanding of the functions of DE miRNAs in sheep testis development, we performed Gene Ontology (GO) and Kyoto Encyclopedia of Genes and Genomes (KEGG) enrichment analyses on the candidate target genes of all DE miRNAs. The GO analysis results are presented in Figure 3. Between M0 and M3, target genes were significantly enriched in categories such as animal organ development, cell morphogenesis, cell part morphogenesis, and Rab GTPase binding (Figure 3A). Between M0 and M6, target genes were significantly enriched in regulation of cellular biosynthetic process, dendrite morphogenesis, tube development, organ morphogenesis, and cell–cell adherens junction (Figure 3B). Between M0 and Y1, target genes were notably enriched in reproductive process, apical junction complex, apical plasma membrane, and ATP binding (Figure 3D). Between M3 and M6, target genes were significantly enriched in cell differentiation, cell morphogenesis involved in differentiation, Wnt signaling pathway, and apical junction complex (Figure 3C). Between M3 and Y1, target genes were enriched in tube morphogenesis, regulation of organelle organization, plasma membrane, Golgi complex, and microtubule binding (Figure 3F). Lastly, between M6 and Y1, target genes were significantly enriched in cell migration, dendrite morphogenesis, organ morphogenesis, and cell–cell junction organization (Figure 3E).

**Figure 4:**
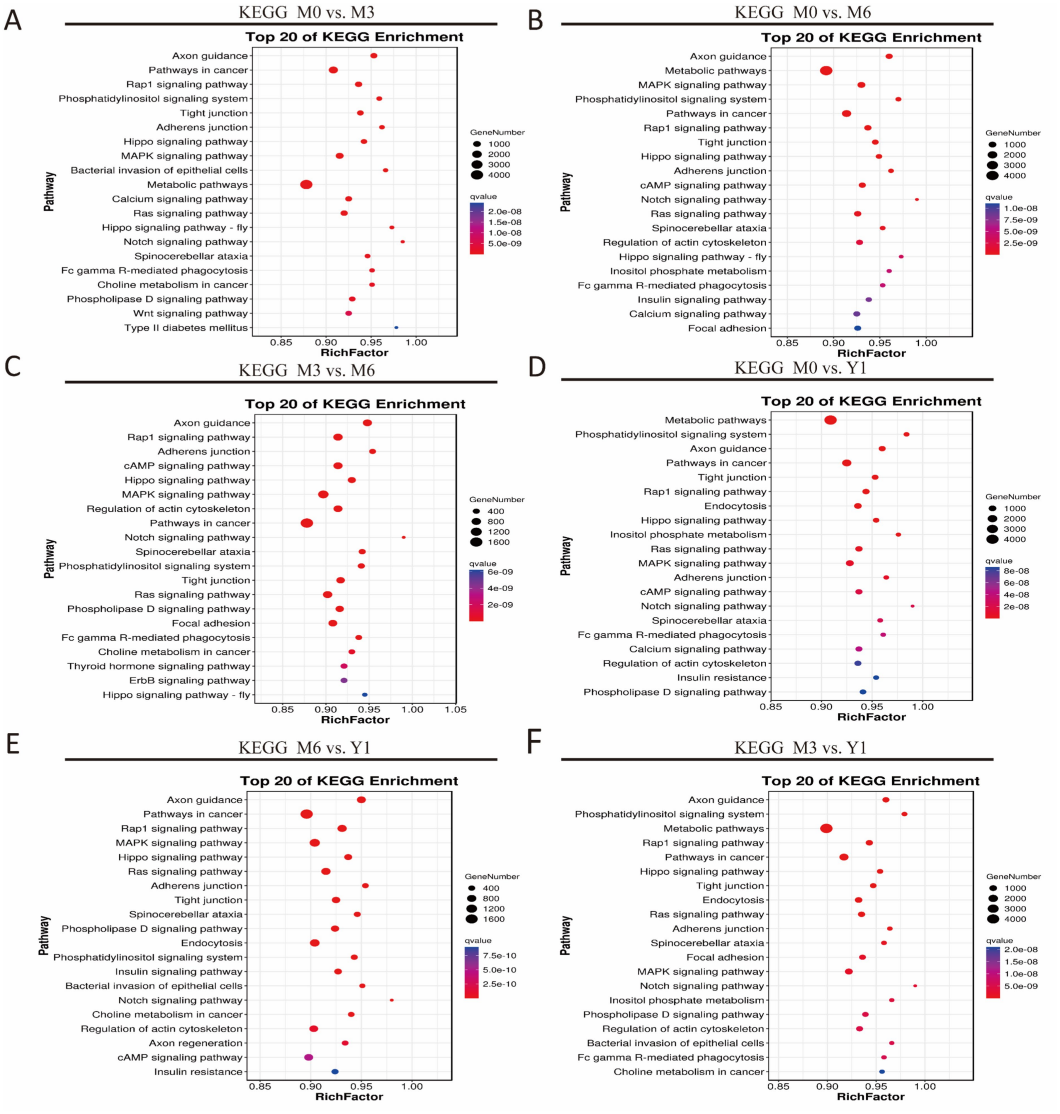
KEGG analysis of target genes of differentially expressed miRNAs. **(A)** M0 vs M3; **(B)** M0 vs M6; **(C)** M3 vs M6; **(D)** M0 vs Y1; **(E)** M6 vs Y1; **(F)** M3 vs Y1; M0 represents 0 months of age, M3 represents 3 months of age, M6 represents 6 months of age, and Y1 represents 1 year of age.

KEGG Pathway Annotation identified significant enrichment of target genes of DE miRNAs in various comparisons. In M0 vs. M3, 30 pathways were enriched, including the Ras, MAPK, Rap1, cAMP, and Notch signaling pathways (Figure 4A). Four key pathways related to testis development were cAMP, MAPK, PI3K-Akt, and Wnt signaling. In M0 vs. M6, target genes were enriched in 30 pathways, such as adherens junction, tight junction, spinocerebellar ataxia, phospholipase D signaling, and endocytosis (Figure 4B). Four testis development-related pathways were MAPK, cAMP, FOXO, and GnRH signaling pathways. Between M3 and M6, 30 pathways were enriched, including Notch, Ras, thyroid hormone, and ErbB signaling (Figure 4C). Five key pathways were Notch, ErbB, endocytosis, Ras, and thyroid hormone signaling.

**Figure 5:**
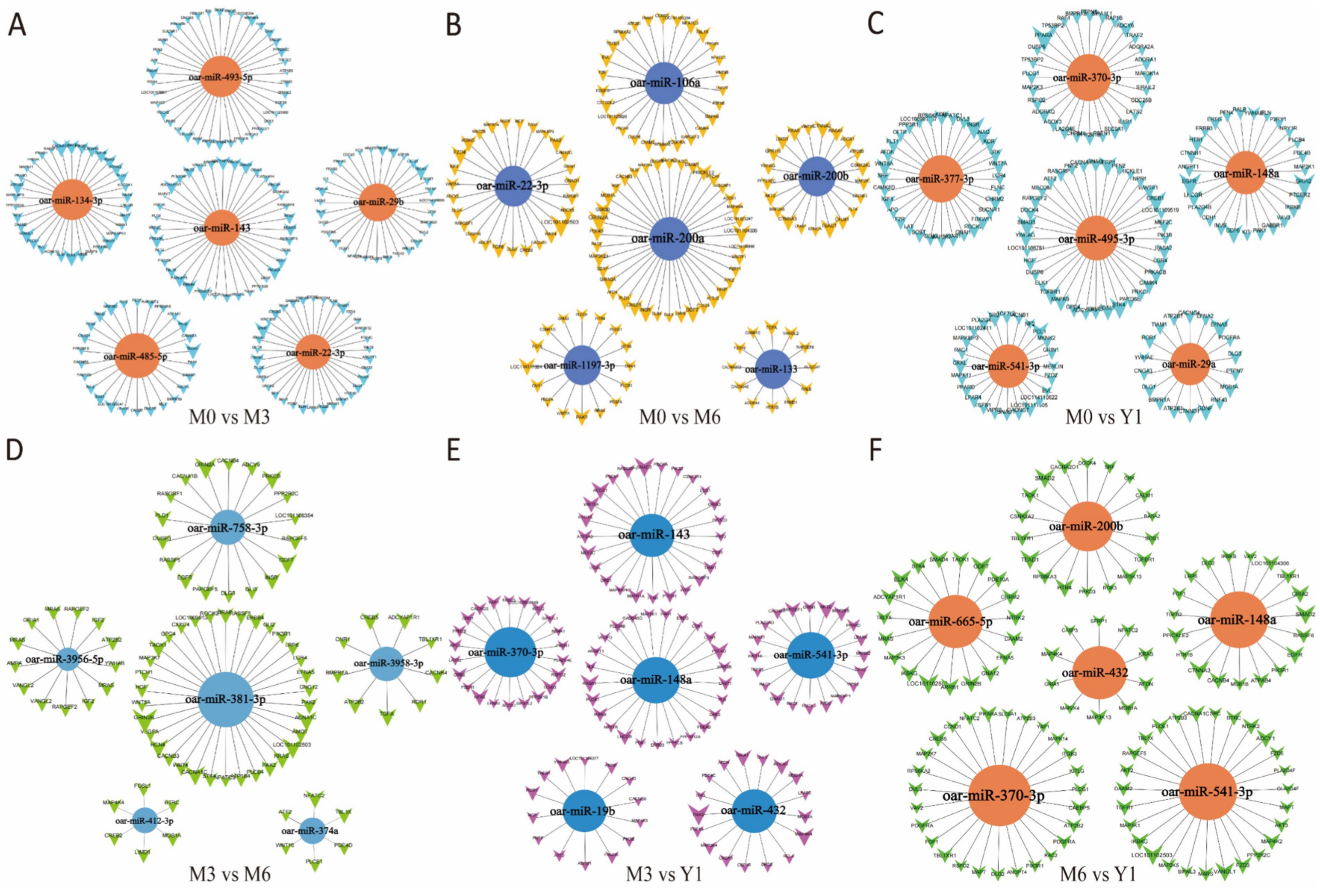
Cluster analysis of differential miRNA co-expression in pairwise comparison groups. **(A)** M0 vs M3; **(B)** M0 vs M6; **(C)** M0 vs Y1; **(D)** M3 vs M6; **(E)** M3 vs Y1; **(F)** M6 vs Y1; M0 represents 0 months of age, M3 represents 3 months of age, M6 represents 6 months of age, and Y1 represents 1 year of age.

In M0 vs. Y1, 49 pathways were enriched, including Notch, Ras, thyroid hormone, and ErbB signaling (Figure 4D). Five testis development-related pathways were FOXO, ErbB, endocytosis, Ras, and thyroid hormone signaling. Between M6 and Y1, 30 pathways were enriched, such as Hippo, adherens junction, cAMP, Notch, and Ras signaling (Figure 4E). Five key pathways were Notch, Hippo, cAMP, endocytosis, and FOXO signaling. Similarly, in M3 vs. Y1, 30 pathways were enriched, including tight junction, endocytosis, Ras, adherens junction, and spinocerebellar ataxia (Figure 4F). Four testis development-related pathways were Ras, MAPK, and cAMP signaling (Figure 5).

### Cluster analysis of miRNA co-expression

We performed co-expression cluster analysis of the differentially expressed miRNAs screened from each comparison group and screened the top 5 miRNAs with degree values. After comparison, the number of miRNA target genes in M0 vs. M3 group, M0 vs. Y1 group and M0 vs. M6 group was higher than that in other groups. Notably, oar-miR-29b, oar-miR-143, oar-miR-370-3p, oar-miR-19b, oar-miR-432, and oar-miR-29b were repeated for different times in the 6 comparison groups. TGF family genes related to testicular development were all regulated by differential miRNAs in the five comparison groups (Figure 5).

### Quantitative RT-PCR validation

**Figure 6:**
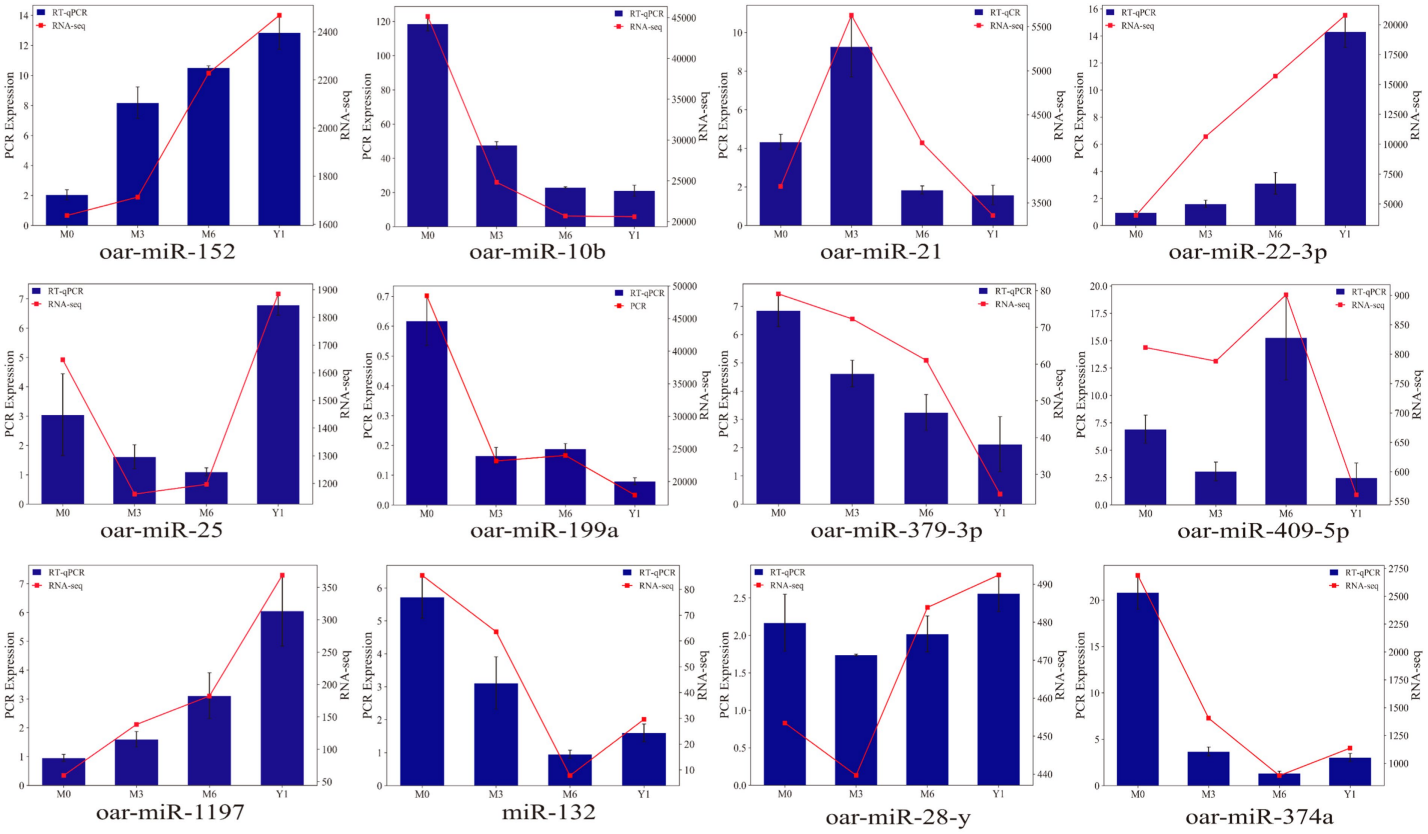
Validation of the expression of differentially expressed miRNAs by qRT-PCR. The blue bar graph represents RT-qPCR, the red broken line represents RNA-seq, the left ordinate is PCR Expression, and the right ordinate is RNA-seq.

Randomly selected 12 known sheep miRNAs, including oar-miR-152, oar-miR-10b, oar-miR-21, oar-miR-22-3p, oar-miR-25, oar-miR-99a, oar-miR-379-3p, oar-miR-409-5p, oar-miR-1179, oar-miR-132, oar-miR-374a, and oar-miR-28-y, were used to validate the RNA-seq data. As shown in Figure 6, the qRT-PCR results exhibited similar expression trends compared to the small RNA-seq results.

## Discussion

The production of sperm and androgens is the primary function of the testis, both of which play a vital role in the entire reproductive system. The differentiation of somatic cells into Sertoli cells initiates male-specific development and guides the development of germ cells toward the spermatogenic lineage (26). Consequently, testicular development and spermatogenesis are crucial processes that affect the reproductive efficiency of male sheep. Additionally, several functional genes have been reported to regulate testicular development and spermatogenesis in sheep (27–29). Recently, miRNAs have been found to regulate biological processes by inhibiting the translation or degradation of target mRNAs (30–32). Since the discovery of miRNAs and their role in gene expression regulation, numerous studies have delved into their involvement in testicular hormone synthesis, cell proliferation, meiosis of haploid spermatocytes, and spermatogenesis (33–35). However, only a few studies have characterized the miRNAs involved in testicular development during early puberty in sheep. In this study, we employed Illumina Solexa technology to sequence small RNAs in testicular tissues from 0-month, 3-month, 6-month, and 1-year-old male sheep, analyzed DE miRNAs, predicted novel miRNAs, and conducted GO enrichment and KEGG pathway analyses of target genes across four miRNA libraries.

The dynamic testicular development of male sheep at 0-month, 3-month, 6-month, and 1-year-old corresponds to the neonatal, pre-pubertal, pubertal, and adult stages, respectively. By comparing the reference genome, we identified 217, 254, 405, 130, 205, and 138 known DE miRNAs in comparisons between M0 vs. M3, M0 vs. M6, M0 vs. Y1, M3 vs. M6, M3 vs. Y1, and M6 vs. Y1, respectively. Notably, more novel DE miRNAs were identified compared to known DE sheep miRNAs, contributing to the identification of key miRNAs in sheep testicular development and enriching the sheep miRBase database. Previous studies have reported that miR-34c, miR-21, and miR-499b regulate the reproductive performance of male mammals, including promoting spermatogonial meiosis, inhibiting Sertoli cell apoptosis, and maintaining the necessary germ cell population of spermatogonial stem cells (SSCs) (36–38). In our study, miR-34c, miR-21, and miR-499b showed significantly higher expression in the Y1 group compared to the M6 group, suggesting their potential involvement in sheep testicular development. Additionally, miR-31-5p downregulation has been detected in the miRNA profiles of seminal exosomes from patients with asthenozoospermia and azoospermia (39) and in non-obstructive azoospermia testes (40), indicating its potential as a predictive marker for azoospermia. Accordingly, miR-155 targets genes involved in MAPK and Wnt signaling pathways, affecting sperm motility (41). Overexpression of miR-10a in testicular germ cells of humans and mice can target and inhibit Rad51 gene expression, leading to meiotic arrest and complete male infertility (42). Similarly, these miRNAs may be associated with sheep testicular development, albeit their specific functions in sheep testes require further exploration and validation.

GO annotation and KEGG pathway analysis can provide a detailed and comprehensive understanding of the functions of DE miRNA target genes from the aspects of cellular components, biological processes, molecular functions and related pathways. GO and KEGG analyses showed that the target genes of DE miRNAs were involved in different biological processes, including growth and development, reproduction, cell proliferation, hormone secretion, and several other metabolic processes. Interestingly, the cAMP, Hippo, MAPK, Wnt, and FOXO signaling pathways are widely recognized to regulate reproduction.

*FOXO1* is mainly expressed in Sertoli cells (43), while *FOXO3a* is expressed in other cells of the testis, such as Leydig cells (44). This cell type specificity allows FOXO family members to regulate the development and function of different cell types in the testis. *FOXO* further regulates testicular development by promoting gonadotropin secretion and testosterone synthesis. The most multifunctional miRNAs controlling target genes in FOXO signaling pathways included oar-miR-19b, oar-miR-200a, oar-miR-369-3p, oar-miR-200b, and oar-miR-493-5p. Each of them regulates key genes in the FOXO signaling pathway such as *FOXO1* and *FOXO6.* The PI3K-Akt signaling pathway directly or indirectly maintains and promotes spermatogenesis by regulating the proliferation, survival, and anti-apoptosis of immature Sertoli cells, mature Sertoli cells, spermatogonial stem cells, and spermatogenic cells (45). In PI3K-Akt signaling pathway, oar-miR-1197-3p, oar-miR-136, oar-miR-199a-3p, oar-miR-410-5p, oar-miR-432 targeted *WT1* gene regulation, *WT1* can not only activate PI3K/Akt and other pathways to inhibit apoptosis, but also plays a crucial role in the assembly and maintenance of fetal testicular cords (46). The Wnt pathway affects the production and maturation of spermatogenic cells by regulating cell proliferation and differentiation. Studies have shown that abnormal activation or inhibition of *Wnt* may affect the normal proliferation and differentiation process of testicular cells, which in turn affects sperm production (47). As a key gene in this pathway, *DOT1L* is co-targeted by DE miRNAs such as oar-let-7f and oar-miR-22-3p. *DOT1L* promotes SSCs self-renewal by catalyzing Histone H3 lysine 79 methylation (H3K79me). It is essential for the maintenance of spermatogenesis (48). The expression of oar-miR-22-3p was found to be significantly changed from M0 to Y1, suggesting that oar-miR-22-3p plays an important role in spermatogenesis and testicular development in sheep. Evidence accumulated over the past few years has highlighted the major functions of MAPK, AMPK, and cAMP signaling pathways during spermatogenesis, with MAPK signaling regulating germ cell proliferation, meiosis, and Sertoli cell proliferation (49). In addition, the AMPK and FOXO signaling pathways influence the proliferation of Sertoli cells (50). Among the MAPK, cAMP, PI3K-Akt, FOXO, Wnt, and AMPK pathways, oar-miR-133, oar-miR-1197-3p, oar-miR-758-3pr, and oar-let-7f are the most potent miRNAs that regulate multiple genes within various testicular development and spermatogenesis-related pathways. Each of these miRNAs plays a crucial role in modulating diverse targets in these signaling cascades. In this study, the downregulation of oar-miR-26a, oar-miR-99a, and oar-miR-148a during the development of sheep testes from neonatal to adult stages suggests their significant contribution to promoting testicular development and spermatogenesis in sheep.

## Conclusion

In summary, we have characterized the miRNA transcriptome across four testicular developmental stages, from birth to adulthood, in the F1 offspring of a cross between Southdown sheep and Hu sheep. A total of 788 known miRNAs and 415 novel miRNAs were identified across the 12 libraries. GO and KEGG pathway analyses of all DE miRNA targets in the libraries revealed significant enrichment in MAPK signaling pathway, Rap1 signaling pathway, Wnt signaling pathway, and FOXO signaling pathway. We believe these results will contribute to a deeper understanding of the roles of miRNAs in sheep testicular development and potentially facilitate the discovery of miRNAs to enhance the reproductive performance of male sheep in the future.

## Data Availability

The data presented in the study are deposited in the NCBI SRA repository, accession number PRJNA1195695.

## Footnotes

^1^ http://www.sanger.a.uk/softare/Rfam

^2^ http://www.mirbase.org/

## Supplementary material

SUPPLEMENTARY TABLE S1 Details of primer sequences of miRNAs used for RT-qPCR.

SUPPLEMENTARY TABLE S2 miRNA Sequence Quality Control.

SUPPLEMENTARY TABLE S3 Read mapping summary of sheep miRNA.

SUPPLEMENTARY TABLE S4 The novel miRNAs and known miRNAs identified in all libraries.

SUPPLEMENTARY TABLE S5 Differentially expressed miRNAs between M0 and M3.

SUPPLEMENTARY TABLE S6 Differentially expressed miRNAs between M0 and M6.

SUPPLEMENTARY TABLE S7 Differentially expressed miRNAs between M0 and Y1.

SUPPLEMENTARY TABLE S8 Differentially expressed miRNAs between M3 and M6.

SUPPLEMENTARY TABLE S9 Differentially expressed miRNAs between M3 and Y1.

SUPPLEMENTARY TABLE S10 Differentially expressed miRNAs between M6 and Y1.
